# Astragaloside IV alleviates heart failure via activating PPARα to switch glycolysis to fatty acid β-oxidation

**DOI:** 10.1038/s41598-017-02360-5

**Published:** 2017-06-02

**Authors:** Zhiwei Dong, Pei Zhao, Ming Xu, Chen Zhang, Wei Guo, Huihua Chen, Jing Tian, Hongchang Wei, Rong lu, Tongtong Cao

**Affiliations:** 1 Institute of Burn Research, Southwest Hospital, State Key Laboratory of Trauma, Burns and Combined Injury, Third Military Medical University, Chongqing, 400038 China; 2 Department of Pathology, Shanghai University of Traditional Chinese Medicine, 1200 Cailun Road, Shanghai, 201203 China; 3 Division of Cardiothoracic Surgery, Baylor college of medicine, Houston, Texas USA

## Abstract

In heart failure (HF), energy metabolism pathway in cardiac muscle changes from fatty acid β-oxidation to glycolysis. However, the exact mechanism is unknown. Sarcoendoplasmic reticulum Ca^2+^α ATPase (SERCA) expression is downregulated and mitochondrial function is reduced in HF, perhaps partly due to a substantially reduced energy supply for excitation–contraction coupling resulting from a lower fatty acid β-oxidation rate. We investigated whether Astragaloside IV can activate peroxisome proliferator-activated receptor alpha (PPARα) to stimulate fatty acid β-oxidation and increase cardiac energy production, improving mitochondrial function and the efficiency of SERCA in HF. In pressure overload-induced HF mice and isolated hypertrophic myocardial cells, fatty acid β-oxidation and heart function were substantially strengthened following Astragaloside IV treatment, as demonstrated by the increased expression of PPARα and SERCA2a. *In vitro*, Astragaloside IV regulated energy metabolism by increasing ATP production and enhancing mitochondrial function, attributable to increased oxygen consumption and slightly increased mitochondrial Ca^2+^ uptake. In HF, Astragaloside IV switched glycolysis to fatty acid β-oxidation, as confirmed by reduced anaerobic glycolysis and an increased oxygen consumption ratio. These results suggest that Astragaloside IV can stimulate fatty acid β-oxidation and improve mitochondrial function, which may present a novel cardioprotective treatment that inhibits the progress of HF.

## Introduction

During the fetal period, glycolysis is the chief energy metabolism pathway in cardiac muscle cells, switching to fatty acid β-oxidation after birth. Reversion to the fetal metabolism is an important change in the progress toward uncompensated heart failure (HF), but the exact mechanism remains unknown. In HF, the substantially reduced energy consumption is insufficient to sustain the strong contractile force; this is accompanied by the depression of ATP generation and decreased phosphorylation of SERCA2a involved in excitation–contraction coupling for cardiac work^1–3^. Although the precise mechanisms are not well understood, improving contractility seems to require the stimulation of ATP generation.

In HF, the primary myocardial energy source changes from normal fatty acid β-oxidation to glycolysis, a reversion to the energy metabolism used during the fetal period^4–6^. Increased glycolytic enzymes and greater lactate accumulation are observed in hypertrophy and HF^5, 7^. It is accepted that fatty acid oxidation provide greater capacity for energy production compared with glucose, reverting the heart back to using fatty acids might be an effective therapeutic approache for treating heart failure^8^. Peroxisome proliferator-activated receptors (PPARs) are members of the nuclear hormone receptor superfamily of ligand-activated transcription factors. There appear to be four PPAR isoforms, referred to as α, β, γ and δ^9^. It has been demonstrated that the expression of PPARα increases considerably after birth to stimulate fatty acid β-oxidation^10^, suggesting that the natural role of PPARα is to regulate lipid mechanism and promote the expression of genes involved in fatty acid β-oxidation^11^. Recently, studies have demonstrated that cardiac dysfunction is deteriorated in PPARα knockout mice in response to chronic pressure overload, and suggested the pathology of heart failure is similar to that of diabetic cardiomyopathy in the heart of myosin heavy chain-PPAR-α transgenic mice^12^. It has already been proved that PPAR-α activation during pressure-overloaded heart failure improved myocardial function and energetics. And it also suggested that activating PPAR-α and modulation of FAO could be a promising therapeutic strategy for heart failure^13, 14^.

Mitochondrial biological function plays a vital role in energy production and cellular metabolic functions^15, 16^, and there has been considerable study of the key role played by mitochondrial Ca^2+^ uptake in increasing ATP synthesis and stimulating Krebs cycle activity, as well as its involvement in the HF progression^17, 18^. A previous study has shown that the maintenance of optimal mitochondrial function is associated to a great extent with Ca^2+^ transport in mitochondrial membranes^16^. Moreover, mitochondrial membrance permeability and the opening of mitochondrial hypertrophic response permeability transition pores also play a key role in mitochondrial biological function. Research has shown that the levels of Ca^2+^ within mitochondria cause mitochondrial permeability transition pores to open, which can result in the loss of the membrane potential, affecting energy metabolism and cell function^19^. In addition, the relationship between Ca^2+^ uptake and PPARα has recently been elucidated: PPARα can regulate mitochondrial Ca^2+^ uptake sites via interacting with PPARγ coactivator 1 (PGC-1) in cardiac tissues^20, 21^. During HF or cardiac hypertrophy, the decline in Ca^2+^ uptake results in a lower rate of ATP production and a decrease in oxidative capacity.

Astragaloside IV (AST) is a herbal remedy which is purified from *Astragalus membranaceus*. It has been shown to have multiple beneficial effects on diabetes through promoting the expression of PPARγ^22, 23^, and it has recently been shown to have a therapeutic action in neurodegenerative disease^24^. It also exhibits substantial inhibitory effect on apoptosis and the oxidative stress on hypertrophic cardiomyocytes *in vivo* and *in vitro* ^25^. However, few studies have investigated the effects of AST on PPARα in HF, which could potentially indicate a new direction for HF treatment. In the present study, we tested the hypothesis that AST would have beneficial effects on HF via stimulating PPARα expression and promoting mitochondrial metabolism to meet the energy consumption of normal excitation–contraction coupling.

## Results

### AST treatment exerted a protective effect by improving the contraction function in a HF model

**Figure 1:**
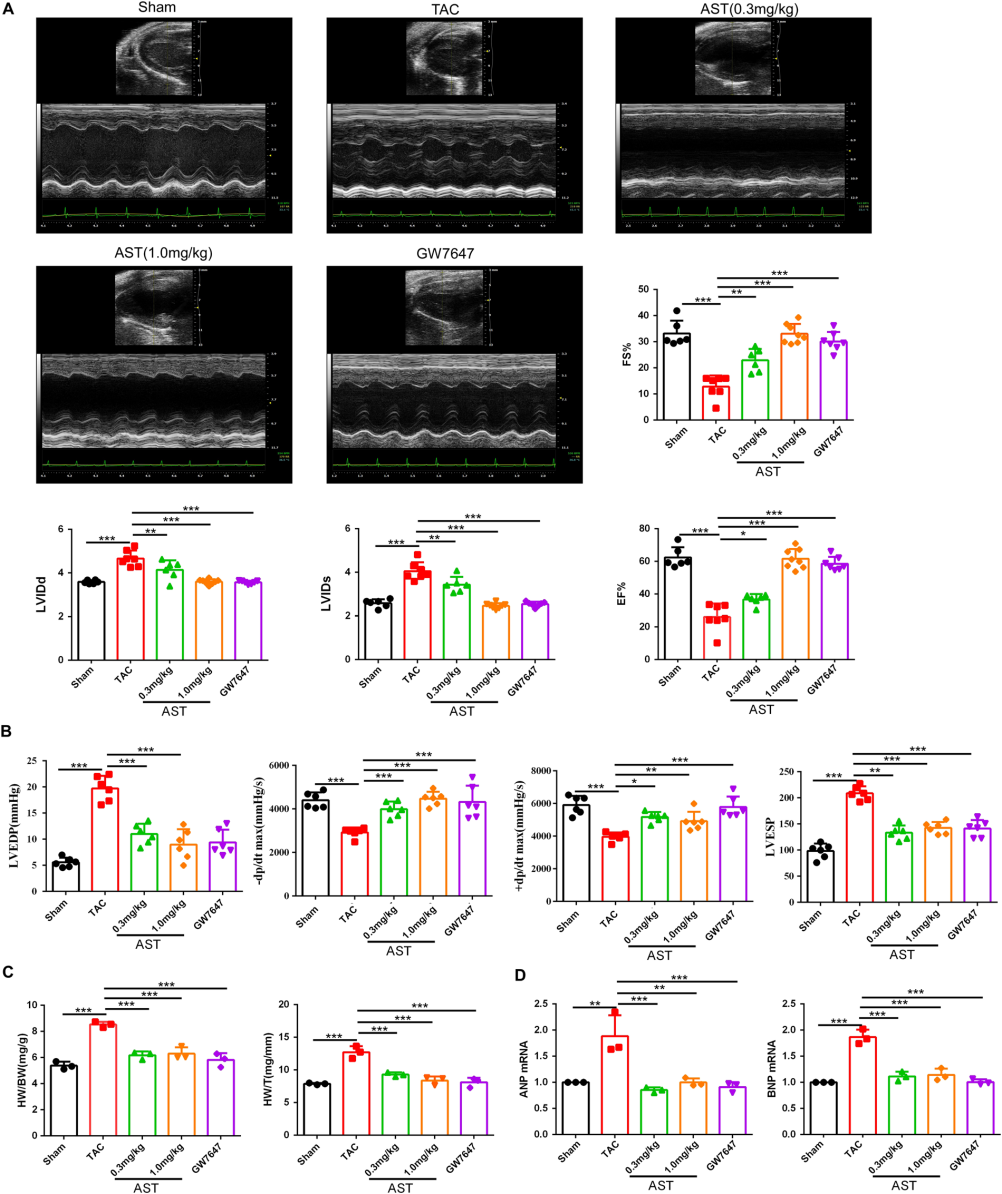
Astragaloside IV (AST) treatment exerted a protective effect that improved the contraction function in transverse aortic constriction-induced heart failure. (**A**) Representative M-mode echocardiography images. EF, ejection fraction; FS, fractional shortening; LVIDs, left ventricular internal systolic diameter; LVIDd, left ventricular internal diastolic diameter. Sham group (n = 6), transverse aortic constriction (TAC) group (n = 8), low dose AST (0.3 mg/kg; n = 9), high dose AST (1.0 mg/kg; n = 6), and GW7647 (1 µg/g; n = 7). (**B**) The calculated hemodynamic parameters are shown as indices of heart function (LVESP, left ventricular end-systolic pressure; LVEDP, left ventricular end-diastolic pressure; ±dP/dt, rate of increase/decrease in maximum pressure). (**C**) Quantitation of heart-to-body weight ratio and heart weight-to-tibia length ratio. (**D**) Quantitative real-time polymerase chain reaction analysis of *ANP* and *BNP* expression. *P < 0.05, **P < 0.01 and ***P < 0.0001. Data are mean ± s.d.

To investigate the potential protective effects of AST in HF, we conducted a study with a mouse model of HF induced by transverse aortic constriction (TAC). TAC mice develop hypertrophy at around 4–6 weeks and HF at around 8–10 weeks, as demonstrated by echocardiography and hemodynamic analysis^24^. In the present study, we confirmed that our TAC mice had notable impairment of systolic and diastolic function at 8 weeks, as shown by notable decreases in fractional shortening, ejection fraction, left ventricular maximum systolic and minimum diastolic velocities, and left ventricular end-diastolic and end-systolic pressures, as well as obvious enlargement of the systolic and diastolic left ventricular internal diameters (Fig. 1A and B). Compared with a sham group, the TAC mice displayed expanded hearts with significantly increased heart-to-body weight ratio (HW/BW) and heart weight-to-tibia length ratio (HW/T) (Fig. 1C) and upregulated expression of the ANP and BNP genes (Fig. 1D). To investigate the effect of AST in TAC-induced HF, one week after TAC surgery the mice were treated with either a low dose (0.3 mg/kg/day) or a high dose (1.0 mg/kg/day) of AST. Compared with the TAC group, the high dose group exhibited clear preservation of systolic and diastolic function, with increased fractional shortening, ejection fraction, left ventricular maximum systolic and minimum diastolic velocities, and left ventricular end-diastolic and end-systolic pressures, as well as attenuation of the systolic and diastolic left ventricular internal diameters (Fig. 1A and B). Decreased HW/BW and HW/T ratios and the lower expression of the *ANP* and *BNP* genes provided further evidence of the protection of AST in TAC-induced HF (Fig. 1C and D). To confirm that TAC-induced HF was a PPARα-dependent process, we used GW7647, a special selective PPARα agonist, to activate the PPARα. Interestingly, GW7647 treatment (1 µg/g/day) also ameliorated heart function and downregulated the expression of *ANP* and *BNP* in TAC-induced HF, indicating that PPARα was closely involved in the progression of HF. This raised the question of whether the protective effect of AST was associated with the activation of PPARα in HF.

### AST accelerated PPARα and PPARα-targeted genetic expression in TAC-induced HF

**Figure 2:**
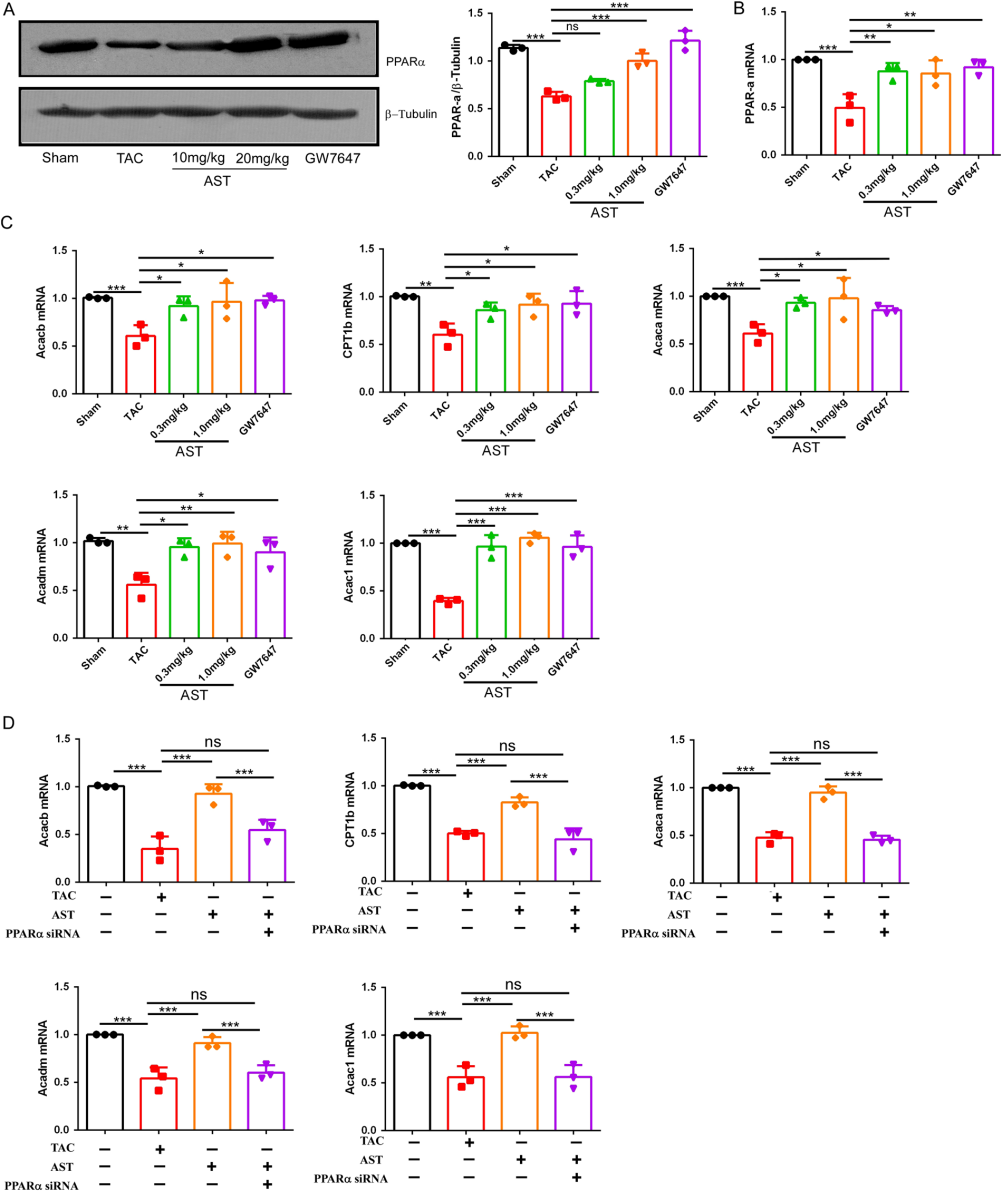
Astragaloside IV modulated PPARα and PPARα-targeted genetic expression in transverse aortic constriction-induced heart failure. (**A**) Western blotting analysis of PPARα expression. *P < 0.05, **P < 0.01 and ***P < 0.0001 (n = 4). The original gels images used for A are shown in Supplementary Fig. S1. (**B**) Quantitative real-time polymerase chain reaction (RT-PCR) analysis of PPARα gene expression. *P < 0.05, **P < 0.01 and ***P < 0.0001 (n = 4). (**C**) and (**D**) Quantitative RT-PCR analysis of the expression of the PPARα-targeted genes Acaca, Acacb, Cpt1b, Acacm, and Acac1. Acaca: acetyl-Coenzyme A carboxylase alpha; Acacb: acetyl-CoA carboxylase beta; Cpt1b: carnitine palmitoyltransferase 1B; Acadm: Acyl-CoA Dehydrogenase, C-4 To C-12 Straight Chain; Acac1: acetyl-CoA carboxylase 1. *P < 0.05, **P < 0.01 and ***P < 0.0001 (n = 5). Data are mean ± s.d.

PPARα has been shown to play a crucial role in the progression of HF and to be closely associated with ATP production; its downregulation leads to a decline in fatty acid β-oxidation. To determine that the effects of AST on PPARα in TAC-induced HF, we examined the expression of PPARα at the mRNA and protein level. Surprisingly, as shown in Fig. 2A and B, the expression of PPARα was significantly reduced in the TAC model, and this effect was suppressed in the high dose AST treatment mice. However, it did not change greatly with low dose AST treatment, as determined by western blot and real-time polymerase chain reaction (PCR) analysis. It seemed that AST has a similar function to GW7647, which were attributable to activating PPARα. Furthermore, given that AST can modulate the expression of PPARα-targeted genes, including *Acaca*, *Acacb*, *Cpt1b*, *Acadm*, and *Acac1*, the expression of these genes was also analysed. Compared with the TAC model group, AST treatment exhibited significant upregulation in the expression of all five of these genes, as detected by real-time PCR (Fig. 2C). To test the effect of AST on PPARα and PPARα targeted gene expression more specifically, we used PPARα siRNA to see if it can block AST induced PPARα gene expression in cardiomyocytes. Surprisingly, as shown in Fig. 2D, the increased expression of PPARα and PPARα targeted gene including *Acaca*, *Acacb*, *Cpt1b*, *Acadm*, and *Acac1* in AST treatment were inhibited by PPARα siRNA (Fig. 2D). Taken together, these results confirmed that AST may specifically upregulate the expression of PPARα and PPARα-targeted genes.

### AST treatment attenuated the decrease in mitochondrial respiration rate and production of ATP in isolated hypertrophic myocardial cells

**Figure 3:**
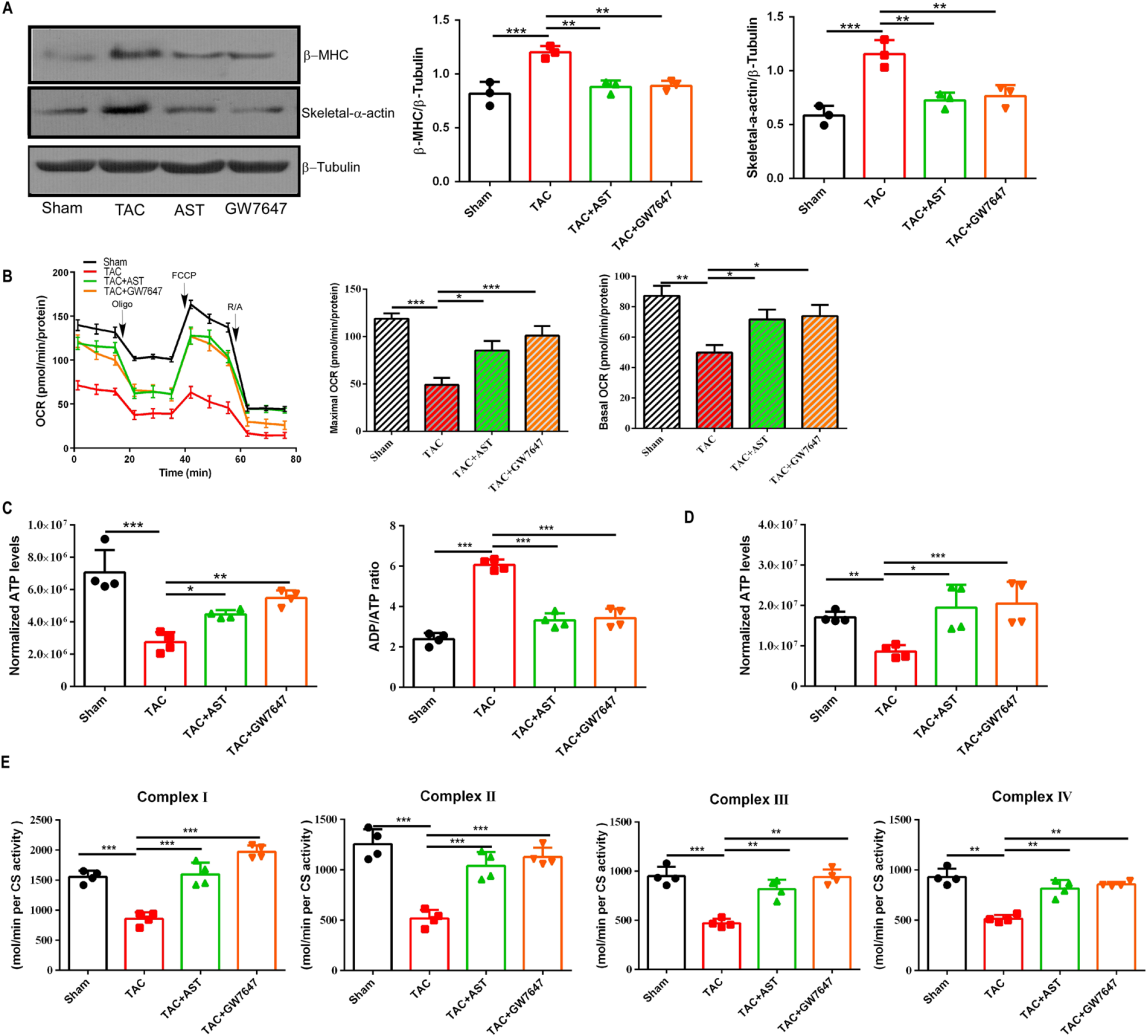
The effect of Astragaloside IV treatment on the mitochondrial respiration rate and the production of ATP in isolated hypertrophic myocardial cells. (**A**) Western blotting analysis of β-MHC and skeletal α-actin expression. The original gels images used for A are shown in Supplementary Fig. S2. (**B**) Oligomycine (0.5 μM), carbonyl cyanide-4-(trifluoromethoxy)phenylhydrazone (FCCP) (1 μM), rotenone and antimycin A (1 μM) were injected sequentially at indicated time points into each well containing isolated myocardial cells. Mitochondrial function was assessed by the basal and maximal cellular oxygen consumption rates (OCRs). The representative time course data for the OCR and aggregated data are shown. (**C**) The ATP level expressed relative to control and the ADP/ATP ratio for each group (n = 4). *P < 0.05, **P < 0.01 and ***P < 0.0001 (n = 5). Data are mean ± s.d. (**D**) The ATP level expressed relative to sham *in vivo*. *P < 0.05, **P < 0.01 and ***P < 0.0001 (n = 3). (**E**) The activity of the ETC complexes I, II, III, and IV in cardiac mitochondria. Activities of ETC complexes normalized to mitochondrial protein(mg), respectively. Data are mean ± s.d.

*In vitro*, we clarified the effects of AST against the hypertrophy induced by TAC in myocardial cells isolated from the adult mice at different time points^26^. As shown in Fig. 3A, the protein expression of hypertrophic marker gene β-myosin heavy chain (β-MHC) and skeletal α-actin were significantly increased in isolated myocardial cells from the TAC group in comparison to the sham group. Conversely, TAC-induced hypertrophic growth was remarkably attenuated by both AST and GW7647 treatment (Fig. 3A). To assess and test the potential of AST for enhancing mitochondrial function in cardiac hypertrophy, basal and maximal mitochondrial respiration rates were measured, represented as the oxygen consumption rate. The hypertrophic myocardial cells showed a significantly decline in basal and maximal respiration rates in comparison to the Sham group (Fig. 3B). AST treatment resulted in enhanced oxygen consumption rates in basal and maximal respiration. Interestingly, GW7647 treatment also showed clear effective protection against the reduction in mitochondrial respiration in the hypertrophic myocardial cells, suggesting that PPARα activation had an effective action on mitochondrial respiration.

To investigate further whether the anti-hypertrophic effect of AST was related to the regulation of cellular energy metabolism, we examined ATP levels in isolated hypertrophic myocardial cells using bioluminescence assay and detected a significant reduction in ATP and the ATP/ADP ratio. The ATP production decreased to 40%. However, the reduction of ATP was notably attenuated by the addition of AST. Furthermore, ATP concentration increased strikingly with GW7647 treatment, with a significant difference compared to hypertrophic myocardial cells (P < 0.001) (Fig. 3C). *In vivo*, we also tested examined ATP levels in tissue, the ATP production was also significantly decreased in TAC model, and this reduction was attenuated by AST high dose treatment (Fig. 3D). Furhermore, to confirm the effect of AST on mitochondrial function, we checked the activity of transport chain complexes (ETC) in isolated mitochondria from the heart tissue. As shown in Fig. 3E, the activity of complexes I, II, III, and IV were significantly declined in TAC group, and this reduction can be attenuated by the AST high dose treatment. Thus, AST markedly alleviated the reduction of oxygen consumption rate and ATP production in isolated hypertrophic myocardial cells and *in vivo*. Taken together, these results indicated that AST may exert anti-hypertrophic effects through regulating mitochondrial function and the production of ATP.

### AST treatment inhibited glycolysis and accelerated fatty acid β-oxidation in isolated hypertrophic myocardial cells

**Figure 4:**
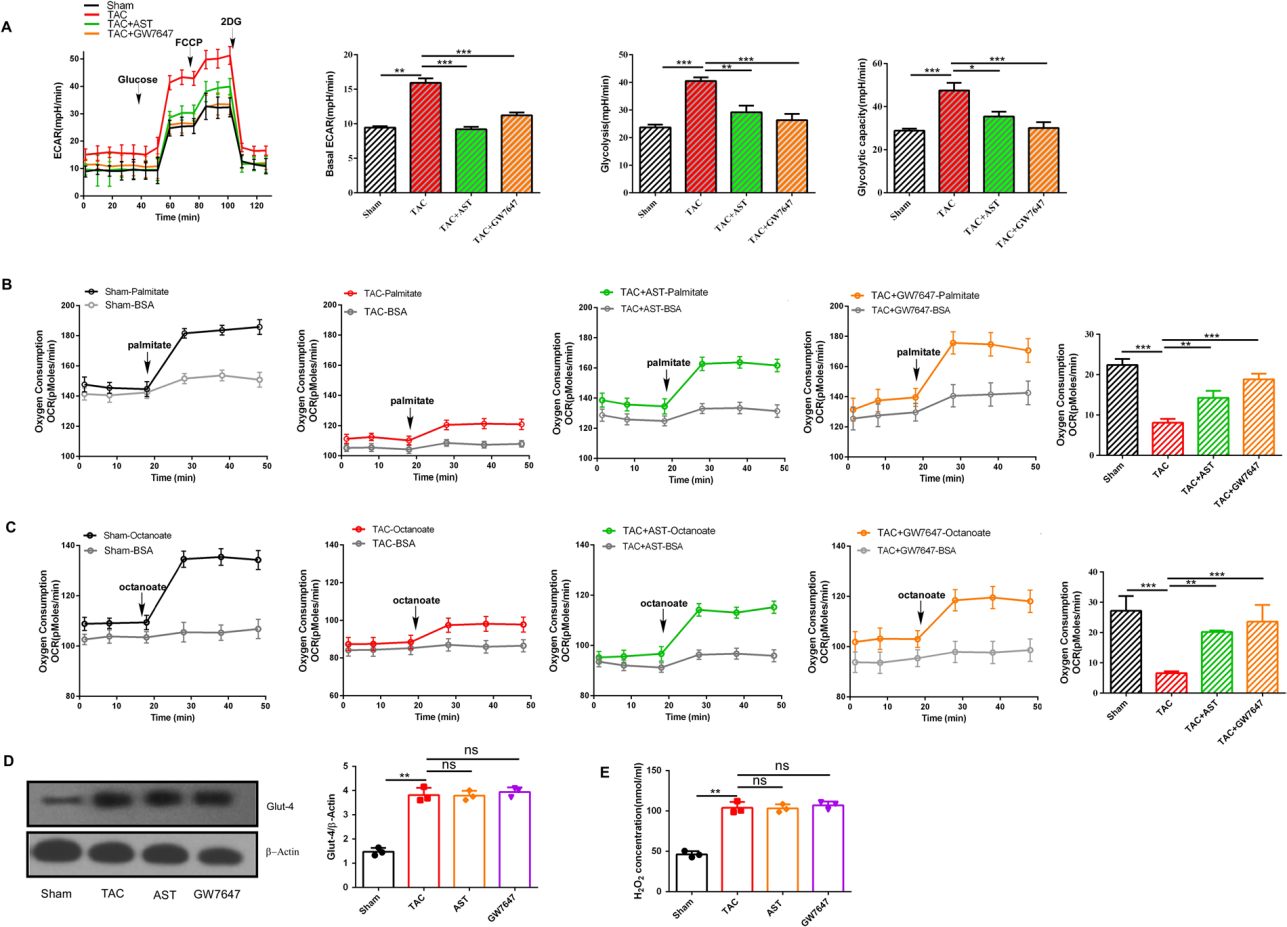
Astragaloside IV treatment inhibited glycolysis and accelerated fatty acid β-oxidation in isolated hypertrophic myocardial cells. (**A**) Extracellular acidification rate measurement trace during the Seahorse glycolysis stress assay, in which the medium bathing the isolated myocardial cells was sequentially injected with 20 mM glucose, 1 μM oligomycin and 2-deoxy glucose (20 mM). Glycolysis rates were calculated by subtracting the normalised extracellular acidification rate values after injection of 2-deoxy glucose (20 mM). (**B**) The kinetic oxygen consumption rate (OCR) response in isolated myocardial cells to the long-chain fatty acid palmitate (150 μM). (**C**) The kinetic OCR response in isolated myocardial cells to the medium-chain fatty acid octanoate (1 mM). Isolated myocardial cells were seeded onto a 24-well XF plate. After incubation for 3 h, the culture media was replaced with XF base medium. Fatty acid oxidation was expressed as OCR. *P < 0.05, **P < 0.01 and ***P < 0.0001 (n = 5). Data are mean ± s.d. (**D**) Western blotting analysis of Glut-4 expression. The original gels images used for A are shown in Supplementary Fig. S5. (**E**) The activity of glucose oxidase was detected by glucose oxidase activity assay kit. *P < 0.05, **P < 0.01 and ***P < 0.0001 (n = 3).

AST was found to have the ability to upregulate the expression of PPARα as well as the PPARα-targeted genes and to regulate the production of ATP (Fig. 3B and C). PPARα is widely considered to be a key transcriptional factor involved in changes to fatty acid oxidation. We therefore evaluated the effect of AST on the glycolysis and fatty acid oxidation of cardiomyocytes. In order to demonstrate the effect of AST on glycolysis in hypertrophic myocardial cells, Seahorse flux analysis was performed, and glycolytic flux and capacity were determined from the change of extracellular acidification rate. Glucose was added to the incubated cells to trigger the glycolytic flux, and then oligomycin was added to switch from glucose flux to lactate, revealing the glycolytic capacity. Not only basal acidification rate but also glucose influx and glycolytic capacity were enhanced in the hypertrophic myocardial cells compared with the sham group, but this enhancement was significantly inhibited by both AST and GW7647 treatment (Fig. 4A). The oxidation of fatty acids was determined by measuring the oxygen consumption rate (OCR) following the injection of fatty acids. Previous studies have shown that cardiac muscle is capable of using mostly fatty acids to meet the high metabolic demand; we therefore injected the incubated cells with the fatty oxidation of long and medium chain fatty acid. Compared with the sham group, the hypertrophic myocardial cells had significantly lower OCR levels following the injection of the various fatty acids (Fig. 4B and C). However, AST treatment substantially enhanced the OCR in hypertrophic myocardial cells, and the cells exposed to GW7647 showed a similar increase (P < 0.01). Together, these findings confirmed that the activated glycolysis and the inhibited fatty acid oxidation were consistent with a reduction in ATP production and the reduced expression of PPARα –target genes in hypertrophic myocardial cells. However, AST treatment prevented the overactivation of glycolysis and the decrease in fatty acid oxidation in hypertrophic myocardial cells.

To further investigate the molecular changes on glucose utiliazation occurred with AST treatment, the protein expression of myocardium-specific glucose transporter 4(Glut-4) and hydrogen peroxide levels from different glucose oxidase were measured. As shown in Fig. 4D and E, the expression of Glut-4 and the hydrogen peroxide concentration were significantly increased in isolated hypertrophic myocardial cells compared with control. Interestingly, the increased expression of Glut-4 and the hydrogen peroxide concentration in isolated hypertrophic myocardial cells weren’t change in AST treatment, indicating that the effect of AST on improving energy metabolism in the mitochondrial levels.

### AST enhanced mitochondrial function in isolated myocardial cells

**Figure 5:**
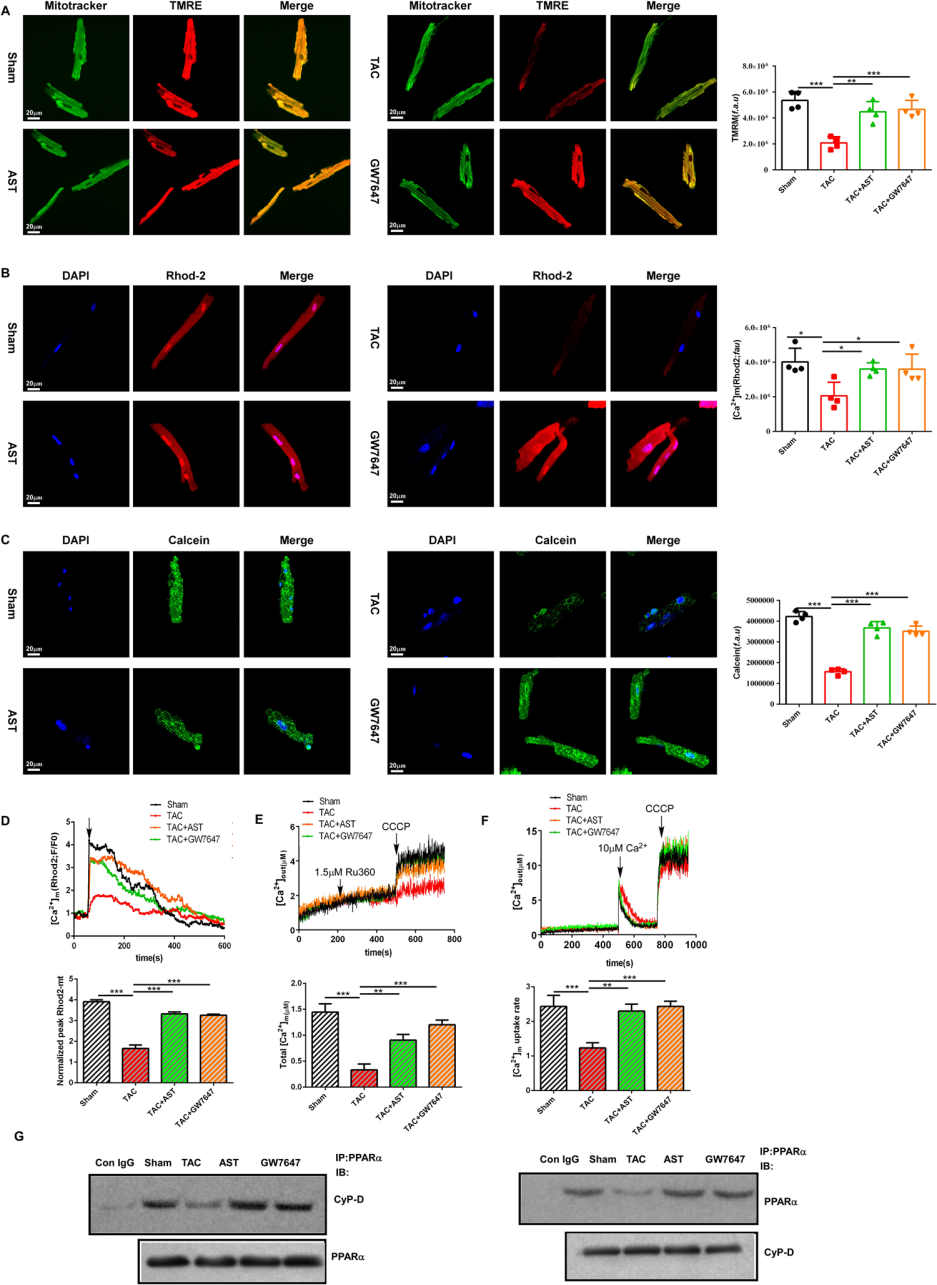
Astragaloside IV enhanced mitochondrial function in isolated myocardial cells. (**A**) Tetramethylrhodamine ethyl ester (TMRE) and Mitotracker double staining were performed to detect mitochondrial inner membrane permeability in isolated myocardial cells. The mitochondrial membrane potential was analysed using TMRE. The bar graph represents the mean ± s.d. of three independent experiments with **P < 0.01 and ***P < 0.0001. (**B**) Rhod-2-specific mitochondrial localisation of Ca^2+^ was determined using fluorescence microscopy. (**C**) Calcein staining of the opening of mitochondrial permeability transition pores was performed as described in the Methods section. The data represent three independent experiments with P < 0.05. Scale bars, 10 μm. (**D**) The capacity for calcium uptake in isolate myocardial cells was determined from mitochondrial Ca^2+^ (Rhod-2 AM) dynamics after stimulation with histamine (100 μM). Data were assayed using confocal microscopy. (**E**) The isolated myocardial cells were permeabilised with 40 µg/ml digitonin in 1.5 ml of intracellular medium along with thapsigargin, Ru360 and CGP37157. The accumulation of basal mitochondrial matrix Ca^2+^ was determined using a multiwavelength excitation dual-wavelength emission fluorimeter (PTI). (**F**) The myocardial cells were pulsed with 20 μM Ca^2+^ and the mitochondrial Ca^2+^ uptake rate was measured using the PTI. **P < 0.01 and ***P < 0.0001 (n = 3). Data are mean ± s.d. (**G**) Cell lysis from each group were immunoprecipitated (IP) with anti-CypD, PPARα. The original gels images are shown in Supplementary Fig. S6. **P < 0.01 and ***P < 0.0001 (n = 3). Data are mean ± s.d.

Mitochondrial Ca^2+^ uptake is a key aspect of mitochondrial function. Several studies have suggested that ATP production and energy metabolism could be enhanced to some extent by mitochondrial Ca^2+^ uptake^27^. To examine whether AST treatment resulted in changes in mitochondrial function, the cells were loaded with Rhod-2 AM+ to stain the calcium in the mitochondria. In addition, it has been shown that the induction of mitochondrial permeability transition can increase mitochondrial membrane permeability, which provides the driving force for ATP production. We therefore used tetramethylrhodamine ethyl ester (TMRE) to indicate mitochondrial inner membrane permeability, and the mitochondrial permeability transition pores were measured by loading the mitochondria with calcein AM. Compared with the sham group, Rhod-2, TMRE and calcein fluorescence intensities were obviously weakened in HF hypertrophic myocardial cells, whereas AST treatment resulted in enhanced fluorescence intensity (Fig. 5A,B and C). GW7647 treatment also exhibited a remarkable enhancement of TMRE, Rhod-2 and calcein fluorescence intensities. We also measured the maximal mitochondrial Ca^2+^ dynamic change after exposure to 10 μm histamine (a G-protein-coupled receptor agonist). Compared with the control myocardial cells, we observed a significant reduction in the amplitude of mitochondrial Ca^2+^ in cells isolated from the TAC mice. In contrast, AST and GW7647 treatment changed the reduction in mitochondrial Ca^2+^ response to histamine in hypertrophic myocardial cells (Fig. 5D). To verify these results, permeabilised myocardial cells in an intracellular matrix (ICM) containing succinate to energise the mitochondria, thapsigargin to block ER Ca^2+^ uptake and Fura-2FF to monitor extramitochondrial changes. We used Ru360 (mitochondrial uniporter inhibitor) and CGP37157 (mitochondrial Na+/Ca2+ exchange inhibiter) to prevent both the influx and efflux of Ca^2+^. Carbonyl cyanide m-chlorophenylhydrazone (CCCP) was added to depolarise the mitochondrial membrane to trigger the release of all mitochondria-stored Ca^2+^. A reduced accumulation of basal mitochondrial matrix Ca^2+^ was observed in the TAC groups, but this decrease was inhibited by AST and GW7647 treatment (Fig. 5E). We then pulsed cells with 10 μM Ca^2+^ to measure the mitochondrial Ca^2+^ uptake. The Ca^2+^ uptake rate with lower in the TAC group than in the sham group (Fig. 5F). Interestingly, AST and GW7647 treatment both showed a higher Ca^2+^ uptake rate than that of the TAC group. Taken together, these results suggest that AST has an effect in promoting mitochondrial function by regulating calcium uptake, mitochondrial basal calcium, membrane potential and permeability transition pore opening, confirming that PPARα activation could be a key target for AST in this process.

Cyclophilin D is a main regulator of mitochondrial permeability transition pore formation. It has been suggested that PPARα is associated with mitochondrial membrane potential and permeability transition pore opening via association with cyclophilin D. Therefore, we next tested whether AST treatment can regulate the interaction bewteen PPARα and cyclophilin D in myocardial cells. As shown in Fig. 5G, the interaction between PPARα and cyclophilin D was significantly decreased in cells isolated from the TAC mice, but this decline was inhibited by AST treatment, indicating that AST can regulate mitochondrial function via regulating the interaction between PPARα and cyclophilin D.

### AST treatment enhanced the expression of Ca^2+^ regulatory proteins in the sarcoplasmic reticulum and inhibited the expression of apoptotic protein

**Figure 6:**
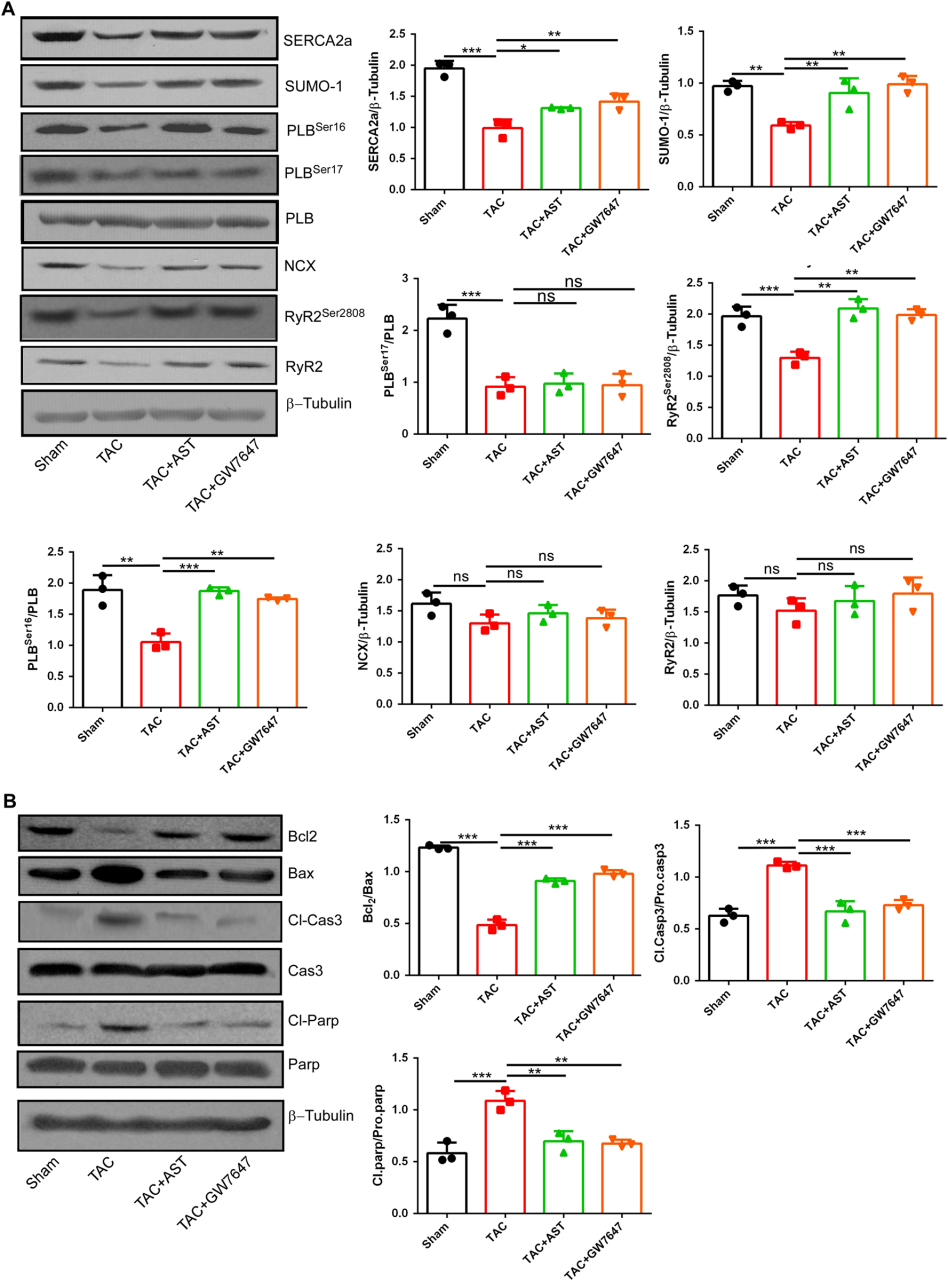
Astragaloside IV treatment enhanced the expression of Ca2+ regulatory proteins in the sarcoplasmic reticulum and inhibited the expression of apoptotic protein. (**A**) Heart tissue fractions were prepared and the collected supernatants containing 50 μg of protein were subjected to western blot analysis and probed with antibodies against SERCA2a, NCX, SUMO1, RyR2, p-RyR2 Ser2808, p-PLB ser16, p-PLB thr17. The data represent three independent experiments. The original gels images are shown in Supplementary Fig. S3. (**B**) Heart tissues were lysed and the proteins subjected to immunoblotting with antibodies against the apoptosis-related proteins Bcl-2, Bax, cleaved caspase-3, caspase-3, cleaved PARP and PARP. α-Tubulin was used as the marker for the cytosolic fractions. The original gels images used for A are shown in Supplementary Fig. S4. *P < 0.05, **P < 0.01 and ***P < 0.0001 (n = 5). Data are mean ± s.d.

The activity of the calcium pump SERCA2a is considerably reduced in heart hypertrophy and HF^28^. This decreased activity severely impacts the contraction and relaxation function in the myocardium. Previous studies have demonstrated that the change in SERCA2a activity during HF substantially contributes to mitochondrial dysfunction. The present study showed the protective effect of AST in mitochondrial dysfunction, and so we investigated whether AST could potentially affect heart function through activating SERCA2a. Compared with the sham group, the expression of SERCA2a in hypertrophic myocardial cells decreased, and this was clearly prevented by AST treatment. To determine whether PPARα was involved in the activation of SERCA2a, we investigated the expression of SERCA2a in GW7647 treatment. This showed that GW7647 treatment significantly enhanced the expression of SERCA2a (Fig. 6A). Recent studies have shown that activation of SUMO1 enhanced the ATP-binding affinity of SERCA2a. In addition, it has been proved that SERCA2a and SUMO1 levels were both reduced in pig models of heart failure and failing human ventricles. Here, we examined the expression level of SUMO1 in cardiac hypertrophy and the change with AST treatment. Consistent with previous reports, we observed the level of SUMO1 to be significantly reduced in hypertrophic myocardial cells. However, the protein expression of SUMO1 clearly increased with both AST and GW7647 treatment (Fig. 6A). Several studies suggest that phospholamban (PLB) is closely regulates SERCA2a activity and thus indirectly controls Ca2+ reuptake to sarcoplasmic reticulum in cardiac contractility. We also checked the phosphorylation levels of the proteins phospholamban (PLB). Compared with the TAC group, a notable elevation in the phosphorylation of PLB Ser16 was observed in AST treatment group and GW7647 treatment group, whereas the expression of phosphorylated PLBthr17 had no change. We further checked the expression of NCX, total RyR2 and phosphorylated RyR at Ser2808. NCX and total RyR2 were slightly increased, although this increase was not statistically significant, whereas the expression of phosphorylated RyR2 at Ser2808 was significantly increased (Fig. 6A). Taken together, these finding suggested that AST had a functional benefit for cardiac contractility partly through increasing the expression of phosphorylated PLB, SERCA2a and phosphorylated RyR2 involved in Ca^2+^ homeostasis. We also observed that AST and GW7647 treatment both significantly abolished apoptosis in comparison to the TAC model group, confirmed by lower expression of the apoptosis-related proteins Bax, cleaved PARP, and cleaved caspase-3, and higher expression of Bcl2 (Fig. 6B).

## Discussion

Fatty acid β-oxidation is the main source of this energy in the adult heart. However, in pathological hypertrophy and HF, the chief energy source reverts to glycolysis, the main energy source for metabolism in the fetal heart, and this impairs myocardial energy production^29^. It is well established that PPARα enhances fatty acid oxidation and upregulates β-oxidation^30^. Various gain or loss of PPARα function in animal models resulted in mixed outcome with either protective or aggravating roles of PPARα in cardiac function. Many metabolic and pathological stress conditions influence cardiac PPARα expression in multiple ways, which are still obscure^31^. Decreased fatty acid oxidation (FAO) is a hallmark of the metabolic remodeling that occurs in the development of cardiac hypertrophy, along with increased glucose utilization. This reduction in FAO has been hypothesized to contribute to contractile dysfunction in cardiac failure. Decreased FAO and increased glycolysis has been repeatedly demonstrated in pathological cardiac hypertrophy^32, 33^. Recent studies demonstrated that a sustained increase in FAO maintaining high rates of FAO could improve cardiac function and energetics during the development of pressure-overload hypertrophy in mice with cardiac-specific deletion of acetyl CoA carboxylase 2 (ACC2)^34^. It is accepted that glucose has the advantage of being more oxygen-efficient compared to FA, but it is dissatisfactory that only 2/3 of the carbon in glucose is oxidized compared to the complete oxidation of FA^34^. Therefore, reliance on carbohydrate metabolism likely represents an energy-deficient state which contributes to contractile dysfunction in heart failure. In this study, we showed that mouse heart function was significantly ameliorated by GW7647 treatment, indicating that this PPARα agonist induced the activation of FAO and improved the contractile function in TAC-induced HF. AST treatment also exhibited a protective function against the development of HF. We proposed that this cardioprotection by AST may result from increasing FAO via PPARα activation. Interestingly, in this study, PPARα was significantly inhibited in TAC-induced hypertrophy and HF, and we found that AST upregulated the expression of PPARα and PPARα-targeted genes such as *Acaca*, *Acacb*, *Cpt1b*, *Acadm*, and *Acac1*. Furthermore, we also found that the increased expression of PPARα and PPARα targeted gene in AST treatment were inhibited by PPARα siRNA. These findings collectively demonstrated that increasing fatty acid oxidation via activating PPARα has the protective effect in TAC-induced HF and also confirmed that AST may specifically upregulate the expression of PPARα and PPARα-targeted genes.

Optimal myocardial bioenergetics rely on efficient ATP synthesis. In HF, however, the main substrate for energy production switches from fatty acids to glucose, resulting in reduced ATP production. In particular, as the process of hypertrophy progresses towards HF, the lower energy synthesis generated by glucose oxidation leads to loss of heart function. It has been reported that the Krebs cycle and mitochondrial respiratory chain is positively associated with cardiac work. The efficiency of ATP utilisation and synthesis are controlled by the activation of the mitochondrial respiratory chain via oxidative phosphorylation. As observed in isolated hypertrophic myocardial cells, the presence of cardiac hypertrophy led to reductions in ATP and the ATP/ADP ratio, resulting in an insufficient overall energy supply for cardiac work. We propose that the switch of fatty acid β-oxidation to become the secondary source of ATP production contributes to the progressive deterioration in heart function in hypertrophy and HF. In support of this, we showed that ATP and the ATP/ADP ratio increased in hypertrophied cardiac cells treated with GW7647 through stimulating PPARα to enhance fatty acid β-oxidation, resulting in a significant decrease in the expression of ANP and BNP genes *in vivo* and the expression of the hypertrophic marker gene β-MHC *in vitro*. We also showed that AST enhanced the production of ATP in hypertrophied myocardial cells, similar to GW7647 treatment. The resulting increased ATP production was responsible for the stimulation of fatty acid oxidation via PPARα in isolated hypertrophic myocardial cells.

The coupling efficiency is the proportion of mitochondrial respiration used for ATP synthesis. Importantly, the upregulation of mitochondrial respiration rates corresponds with increasing mitochondrial ATP production and oxygen consumption^35^. In this study, GW7647 treatment improved mitochondrial function in hypertrophic cells as evidenced by the upregulated mitochondrial respiration rates and increased oxygen consumption. In addition, AST treatment resulted in an improvement in mitochondrial function in Angiotension II induced cardiac hypertrophy. It is possible that AST treatment in hypertrophied myocardium improves mitochondrial function via stimulating PPARα; this is supported by the present observation that AST increased the expression of PPARα and PPARα-targeted genes.

Mitochondria take up Ca^2+^ from the cytoplasm, which is particularly crucial for maintaining the normal mitochondrial membrane potential and Ca^2+^ buffering capacity^36^. It has been demonstrated that the impairment of mitochondrial Ca^2+^ uptake may lead to a change in mitochondrial morphology^37^. Mitochondrial Δψ of ~180 mV (negative inside) across the mitochondrial inner membrane is the main driving force for mitochondrial Ca2+ entry. It enables mitochondria to take up Ca2+ from strategically located microdomains of high Ca2+ near the plasma membrane and further induce Ca2+-signaling events. As Ca2+ enters the mitochondria, it is partially buffered through the formation of calcium phosphate complexes. In HF, mitochondrial Ca^2+^ uptake is seriously disturbed, and this may be closely associated with a negative impact on energy production for cardiac work^38^. It has been suggested that mitochondrial Ca2+ uptake in individual mitochondrial can be stimulated by ATP. In current study, we demonstrated that the activation of PPARα can promote the production of ATP^39^. In support of this, our results showed reduced mitochondrial Ca^2+^ uptake in isolated hypertrophic myocardial cells. As an insufficient energy supply could contribute to the impairment of mitochondrial Ca^2+^ uptake, we suggest that the upregulation of PPARα could increase the content and uptake of the mitochondrial Ca^2+^. Typically, GW7647 or AST treatment reverses the impairment of mitochondrial Ca^2+^ uptake through stimulating PPARα in the hypertrophic myocardium and thereby enhancing fatty acid β-oxidation.

Transient opening of the pore is important for regulating mitochondrial homeostasis. Normal mPTP sensitivity to stimuli for opening plays a critical role in maintaining normal physiological metabolic function. Cyclophilin D is a critical regulator of the mPTP and directly binds to pore constituents, including ANT and PPARα to facilitate opening^40^. We found that PPARα association with cyclophilin D was significantly decreased in cells isolated from the TAC mice, but this decrease was inhibited by AST treatment. The results confirm that AST can regulate mitochondrial membrane potential and permeability transition pore opening via regulting the interaction between PPARα and cyclophilin D.

Accumulating studies support the important role played by crosstalk between the sarcoplasmic reticulum (SR) and cardiac mitochondria in maintaining normal cardiac excitation contraction coupling^41^. Indeed, mitochondria-derived ATP is an indispensable fuel to support Ca^2+^ cycling in the SR, driving the release of Ca^2+^ from the SR to cytoplasm and SERCA-mediated Ca^2+^ uptake back to the SR. It has been proposed that the decreased SERCA activity in HF may have a negative impact on heart contractility. As mitochondrial ATP production is a central component for matching energy supply and demand, it is reasonable that enhancing mitochondrial ATP production would promote the activation of SERCA, which prevents the serious impairment of cardiac contractility in HF^42^. Several studies have demonstrated that the levels and activity of SERCA2a in the SR parallel those of SUMO1^43^. We found that SERCA2a and SUMO1 were downregulated in hypertrophic myocardial cells which was prevented in AST- or GW764-treated groups. Therefore, we propose that the beneficial effect of AST in our studies was associated with the enhancement of ATP production and promotion of the activation of SERCA2a and SUMO1 via activation of PPARα in the hypertrophied heart.

PLB is a well-known major regulator of SERCA2a activity. Binding of PLB to SERCA2a inhibits the pump’s affinity for Ca2+, whereas phosphorylation of PLB suppresses this inhibition^44^. In HF, expression of PLB appears unchanged, whereas phosphorylation of PLB is obviously decreased. Interaction of PLB and SERCA2a is disrupted by phosphorylation of PLB at ser16 or thr17^45^. Phosphorylated PLB ser16 is the cAMP-dependent protein kinase(PKA)-dependent phosphorylation site. There was a general agreement that CaMKII phosphorylates PLB at Thr-17^46^. PKA-dependent RyR phosphorylation has been reported to increase RyR open probability at the single channel level^47^. Moreover, cAMP produced by different adenylyl cyclases (ACs) has different cardiac effects: AC5-derived cAMP is detrimental, whereas AC6-derived cAMP is protective in heart failure^48^. Together, these lines of evidence suggest that different cyclic nucleotide signaling modules have distinct, unique, and even opposing physiological roles. In present study, we found that the phosphorylation of PLB Ser16 and thr17 were significantly decreased in TAC group. Interestingly, compared with TAC group, a notable elevation in the phosphorylation of PLB Ser16 was observed in AST treatment group, whereas the expression of phosphorylated PLBthr17 had no change. Moreover, the expression of phosphorylated RyR2 at Ser2808 was significantly increased after AST treatment. Taken together, these finding confirmed that AST had a functional benefit for cardiac contractility partly through increasing the expression of phosphorylated PLB Ser16, SERCA2a and phosphorylated RyR2 at Ser2808 involved in Ca2+ homeostasis.

## Conclusion

In this study, we observed the important effect of AST on energy metabolism and mitochondrial function in HF. Our study revealed underlying molecular mechanisms that involved the mediation of PPARα expression and regulation of oxygen consumption and calcium uptake in the mitochondria. Most importantly, the switch in energy metabolism from glycolysis to fatty acid β-oxidation could represent a promising therapeutic action of AST for HF.

## Methods

### Animal models

All animals were maintained and handled in accordance with the guideline of the National Institutes of Health. The animal experiments were approved by the Institutional Animal Care and Use Committee of Shanghai University of Traditional Chinese Medicine. Adult male C57 mice (22–25 g, 8 weeks) were used in this study. Pressure overload was induced by TAC in mice at 8–10 weeks, as described previously. In brief, a 6.0 silk suture was placed between the innominate and left carotid arteries, Two loose knots were tied around the transverse aorta and a piece of a 27^1^/_2_-gauge needle was placed to the transverse aorta; The mice in the sham group were subjected to the same surgery but without constriction in order to control variables. One week later, AST(Sigma-Aldrich, St. Louis, MI, USA) was injected intraperitoneally twice daily at either a low dose (0.3 mg/kg/day) or a high dose (1.0 mg/kg body weight/day). Both the model and sham groups were given a gavage of distilled water at the same volume as the drug. Eight weeks later, the mice were anaesthetized with ketamine, and the cardiac dimensions and function were analyzed using 7.5-MHz pulse-wave Doppler echocardiography (IE33, Philips). The mice were then killed by decapitation and their hearts gathered for further evaluation.

### Realtime PCR

QRT-PCR was carried out using a cDNA Synthesis Kit and the SGExcel UltraSYBR Mixture (Termo Scientifc, Fremont, USA). Te sequences of primers used were as follows:

PPARa: AGGGGGACTGCATAGTTTGTC (forward), TTCCGGCCATACACAAGGT (reverse);

cpt1b: CCCTCATGGTGAACAGCAAC (forward), AGTTTGCGGCGATACATGA (reverse);

18s: TCAAGAACGAAAGTCGGAG (forward), GGGACATCTAAGGGCATCAC (reverse);

Acaca: CTGGGACAAAGAACCATCCA (forward), ATAATCTGGATGCCCCCAAG (reverse);

Acacb: CCGAGTTTGTCACTCGGTTT (forward), GCATACACTTGACCGCAGC (reverse);

Acadm: AGCTCTAGACGAAGCCACGA (forward), TGAGCCTAGCGAGTTCAACC (reverse);

Acac1: AACGTCTGGACTCCGGTTCT (forward), CGGGTACTCCCACATGTACC (reverse);

ANP: CAGGCATATTGGAGCAAATCC (forward), CCTCATCTTCTACCGGCATC (reverse);

BNP: CTGAAGGTGCTGTCCCAGAT (forward), CCTTGGTCCTTCAAGAGCTG (reverse);

### Echocardiography

Heart function was evaluated in the 10th week after surgery using a Siemens Acuson™ SC2000 high-frequency ultrasound system (Siemens, Inc., Berlin, Germany). The mice were anaesthetized with inhaled isoflurane and their ejection fraction, fractional shortening, and systolic and diastolic left ventricular internal diameters were measured and analyzed using the M-mode.

### Haemodynamic measurement

Following 9 weeks of treatment, hemodynamic tests were performed after the echocardiography evaluation. Pressures were tested using a 1.4-Fr micromanometer-tipped catheter (Millar Instruments), a pressure transducer (TCB-500, Millar Instruments), and a Power Labo System (AD Instruments), and the left ventricular maximum systolic and minimum diastolic velocities and left ventricular end-diastolic and end-systolic pressures were measured.

### Western blotting analysis

The heart tissues were homogenized with cold lysis buffer. Western blotting was performed as previously described^49^. The homogenates were separated on SDS-PAGE and transferred to polyvinylidene difluoride membranes. After blotting, the membranes were probed with skeletal α-actin, mouse, SUMO1, mouse anti-Bcl2, Bax, cleaved caspase-3, caspase-3, cleaved PARP and PARP antibodies (all from Cell Signaling Technologies, Beverly, MA, USA), β-MHC, PPARA, NCX, anti-PLB, (Abcam, Cambridge, MA, USA), Anti-Ryanodine Receptor 2(alomone labs).

### Langendorff perfusion of isolated mouse hearts

The mouse hearts in the 10th week were attached to the Langendorff aPPARatus, with a modified Krebs–Henseleit buffer used as the perfusate (118.5 mM NaCl, 25.0 mM NaHCO_3_, 4.75 mM KCl, 1.18 mM KH_2_PO_4_, 1.27 mM MgSO_4_, 11.0 mM d-glucose and 1.4 mM CaCl_2_). Collagenase II-digested isolated myocardial cells were incubated in gradually increasing Ca^2+^ concentrations. Cells were seeded on 6-well plates precoated with laminin. After incubation for 3 h, the culture media was changed.

### Real-time fluorescence analysis under confocal microscopy

The mitochondrial membrane potential was measured using TMRE (Molecular Probes, Eugene, OR, USA) according to the manufacturer’s protocol. Further counterstaining with MitoTracker Green (200 nM) was used to identify that the TMRE was specifically present in the mitochondria. To detect mitochondrial calcium levels, primary cultures isolated from adult mice and plated on six-well plate were incubated with 2.5 μM Rhod-2 AM (ThermoFisher Scientific, Waltham, MA, USA) for 40 min, then incubated with DAPI for 10 min at room temperature. The change in the opening of mitochondrial permeability transition pores was determined using calcein. The cells were stained with calcein (ThermoFisher Scientific, Waltham, MA, USA) and cocl2 (Sigma-Aldrich, St. Louis, MI, USA) together for 20 mins at room temperature. Fluorescence images were obtained by confocal fluorescent microscopy using a Zeiss LSM510-META laser scanning confocal microscope (Carl Zeiss, Thornwood, NY, USA).

### Mitochondrial Ca^2+^ dynamics

Cardiomyocytes were isolated and placed on laminin-coated 25-mm glass coverslips for 4 h and stained with 2 μM rhod-2/AM (50 min). After 20 min, the stained cells were used for the confocal analysis. After 1 min of baseline recording, an agonist (histamine, 100 μM; Sigma-Aldrich) was added, and confocal images were recorded every 3 s (510 Meta; Carl Zeiss, Thornwood, NY, USA) at 488- and 561-nm excitation using a 40× oil objective to monitor cytoplasmic and mitochondrial Ca^2+^ dynamics simultaneously. The images were analysed and quantified using ImageJ (National Institutes of Health, Bethesda, MD, USA).

### Ca^2+^ uptake and total mitochondrial Ca^2+^ concentration in the permeabilised cell system

As described previously^19^, after collection and being washed in phosphate-buffered saline without Ca^2+^, 4 × 10^6^ cells were permeabilised with 40 µg/ml digitonin in 1.5 ml of intracellular medium (ICM) (120 mM KCl, 10 mM NaCl, 1 mM KH_2_PO_4_ and 20 mM HepesTris, pH 7.2) containing 2 µM thapsigargin (Sigma-Aldrich), 5 mM succinate (Sigma-Aldrich) and 0.5 μM Fura-FF (Life Technologies, Grand Island, NY). Thapsigargin, succinate and Fura-2FF were used to block the SERCA pump, energise the mitochondria and mark the cytosolic Ca^2+^ separately. After 20 s of data recording, Ca^2+^ (10 μM) was added at 500 s and CCCP (2 mΜ) was added at 800 s. For the total mitochondrial Ca^2+^ concentration, different cells were permeabilised with 40 µg/ml digitonin in 1.5 ml of ICM along with SERCA blocker, thapsigargin (2 µM), Ru360 (1.0 µM; Calbiochem, San Diego, CA, USA) and CGP37157 (10 µM; Sigma-Aldrich). At 500 s, CCCP (2 mΜ; Sigma-Aldrich) was added to depolarise the mitochondrial membrane to trigger the release of all mitochondria-stored Ca^2+^. Fluorescence was measured in a multiwavelength excitation dual-wavelength emission fluorimeter (DeltaRAM, Photon Technology International, Edison, NJ, USA).

### Glycolysis stress test and fatty acid oxidation assay

Isolated myocardial cells were seeded onto a 24-well extracellular flux (XF) plate. After 3 h of incubation, the culture media was replaced with XF base medium. Glycolytic flux and capacity were detected by measuring the extracellular acidification rate under specific conditions followed by the sequential addition of glucose (20 mM) (Sigma-Aldrich), oligomycin (1 μM) (Sigma-Aldrich) and 2-deoxy glucose (20 mM) (Sigma-Aldrich).

For the fatty acid oxidation assay, the culture medium was change to the base medium supplemented with 5.5 mM glucose and 50 µM carnitine (Sigma-Aldrich). The long-chain fatty acid palmitate (150 μM), medium-chain fatty acid octanoate (1 mM) were injected to stimulate the maximal OCR response.

### Oxygen consumption

The oxygen consumption rate was measured at 37 °C in an XF96 extracellular analyser (Seahorse Bioscience) as described previously^19^. Isolated myocardial cells were incubated for 3 h and then washed with XF base medium. The assay medium was pre-equilibrated in an incubator at 37 °C without CO_2_ for 1 h. The OCR was measured under basal conditions followed by the sequential injection of oligomycin (0.5 µM), FCCP (1 µM), and finally rotenone and antimycin A (1 µM). The OCR was measured after each injection.

### Cellular ATP Measurement

ATP levels in isolated myocardial cells were determined using a colorimetric ATP Assay Kit (Promega, Madison, WI, USA) according to the manufacturer’s instructions. The results were normalised to the protein levels.

### Mitochondrial isolation

Heart was quickly removed and mitochondria were isolated by differential centrifugation in a Percoll gradient. Briefly, the heart was homogenized manually in a glass homogenizer and centrifuged at 400 g. The supernatant was centrifuged at 9000 g. Centrifugations were carried out during 10 min at 4 °C. Mitochondrial protein concentration was measured.

### Measurement of the Activity of the ETC Complexes

Mitochondria were extracted from homogenizing heart in cold homogenization medium (250 mM sucrose, 5 mM Tris-HCl, 2 mM EGTA)at 4 °C. The homogenate was centrifuged at 760 × g for 10 min at 4 °C. The supernatant was then centrifuged at 8,740 g at 4 °C. The pellet was resuspended in homogenization medium, transfer to sucrose Percoll gradient, and centrifuged at 8,460 × g at 4 °C. Purified mitochondria were washed three times in homogenization buffer. Rotenone-sensitive NADH quinone reductase as substrate assesses ComplexI, malonate-sensitive succinate quinone dichlorophenol indophenol (DCPIP) reductase as substrate assess ComplexII, Complex III activity was determined as antimycin-sensitive quinol cytochrome c reductase, Cytochrome c oxidase (complex IV) was measured as the oxidation of reduced cytochrome c cyanide-sensitive cytochrome c oxidase. All measurements were performed at 37 °C. Protein levels were determined by the method of Bradford with bovine serum albumin as a standard.

### Glucose oxidase activity assay

Glucose oxidase activity assay kit (Catalog# MAK097-1KT, Sigma Aldrich Inc.) was used to measure the activity of glucose oxidase.

### Coimmunoprecipitation

Protein samples were incubated with anti- PPARα or anti-CypD antibodies overnight at 4 °C, and the immuno- precipitates were harvested by protein A/G-agarose beads (Santa Cruz Biotechnology, Santa Cruz, CA). The immunoprecipitated complexes were washed and then subjected to SDS-PAGE, followed by immunoblotting using antibodies for CypD and PPARα (Cruz Bio-technology).

### Statistics

Statistical analysis was performed using Student’s t test, one-way analysis of variance (ANOVA) and the least significant difference (LSD) test. The data are presented as the mean ± standard deviation (SD) of three independent experiments for each group. All tests were two tailed, and the level of significance was set at P < 0.05.

## Electronic supplementary material

Supplementary data

## References

[1] Huss JM, Kelly DP (2005). Mitochondrial energy metabolism in heart failure: a question of balance. The Journal of clinical investigation.

[2] Doenst T, Nguyen TD, Abel ED (2013). Cardiac metabolism in heart failure: implications beyond ATP production. Circulation research.

[3] Murray AJ, Anderson RE, Watson GC, Radda GK, Clarke K (2004). Uncoupling proteins in human heart. Lancet (London, England).

[4] Kantor PF, Lucien A, Kozak R, Lopaschuk GD (2000). The antianginal drug trimetazidine shifts cardiac energy metabolism from fatty acid oxidation to glucose oxidation by inhibiting mitochondrial long-chain 3-ketoacyl coenzyme A thiolase. Circulation research.

[5] Stanley WC, Recchia FA, Lopaschuk GD (2005). Myocardial substrate metabolism in the normal and failing heart. Physiological reviews.

[6] Wallhaus TR (2001). Myocardial free fatty acid and glucose use after carvedilol treatment in patients with congestive heart failure. Circulation.

[7] Sambandam N, Lopaschuk GD, Brownsey RW, Allard MF (2002). Energy metabolism in the hypertrophied heart. Heart failure reviews.

[8] Arumugam S, Sreedhar R, Thandavarayan RA, Karuppagounder V, Watanabe K (2016). Targeting fatty acid metabolism in heart failure: is it a suitable therapeutic approach?. Drug discovery today.

[9] Gilde AJ (2003). Peroxisome proliferator-activated receptor (PPAR) alpha and PPARbeta/delta, but not PPARgamma, modulate the expression of genes involved in cardiac lipid metabolism. Circulation research.

[10] Minnich A, Tian N, Byan L, Bilder G (2001). A potent PPARalpha agonist stimulates mitochondrial fatty acid beta-oxidation in liver and skeletal muscle. American journal of physiology. Endocrinology and metabolism.

[11] Verreth W (2006). Peroxisome proliferator-activated receptor-alpha, gamma-agonist improves insulin sensitivity and prevents loss of left ventricular function in obese dyslipidemic mice. Arterioscler Thromb Vasc Biol.

[12] Linz W (2009). The peroxisome proliferator-activated receptor-alpha (PPAR-alpha) agonist, AVE8134, attenuates the progression of heart failure and increases survival in rats. Acta pharmacologica Sinica.

[13] Kaimoto S (2017). Activation of PPAR-alpha in the early stage of heart failure maintained myocardial function and energetics in pressure-overload heart failure. American journal of physiology. Heart and circulatory physiology.

[14] Kodde IF, van der Stok J, Smolenski RT, de Jong JW (2007). Metabolic and genetic regulation of cardiac energy substrate preference. Comparative biochemistry and physiology. Part A, Molecular & integrative physiology.

[15] Tarasov AI (2013). Frequency-dependent mitochondrial Ca(2+) accumulation regulates ATP synthesis in pancreatic beta cells. Pflugers Archiv: European journal of physiology.

[16] Kaufman RJ, Malhotra JD (2014). Calcium trafficking integrates endoplasmic reticulum function with mitochondrial bioenergetics. Biochimica et biophysica acta.

[17] Cabrera-Orefice A (2015). The Saccharomyces cerevisiae mitochondrial unselective channel behaves as a physiological uncoupling system regulated by Ca2+, Mg2+, phosphate and ATP. Journal of bioenergetics and biomembranes.

[18] Zavodnik IB (2016). [Mitochondria, calcium homeostasis and calcium signaling]. Biomeditsinskaia khimiia.

[19] Mallilankaraman K (2012). MCUR1 is an essential component of mitochondrial Ca2+ uptake that regulates cellular metabolism. Nature cell biology.

[20] Viswakarma, N. *et al*. Coactivators in PPAR-Regulated Gene Expression. *PPAR research* **2010**, doi:10.1155/2010/250126 (2010). 10.1155/2010/250126 PMC2929611 20814439

[21] Finck BN (2007). The PPAR regulatory system in cardiac physiology and disease. Cardiovasc Res.

[22] Yuan W (2008). Astragaloside IV inhibits proliferation and promotes apoptosis in rat vascular smooth muscle cells under high glucose concentration *in vitro*. Planta medica.

[23] Zhang ZG (2012). Astragaloside IV prevents MPP(+)-induced SH-SY5Y cell death via the inhibition of Bax-mediated pathways and ROS production. Molecular and cellular biochemistry.

[24] Holzem, K. M., Marmerstein, J. T., Madden, E. J. & Efimov, I. R. Diet-induced obesity promotes altered remodeling and exacerbated cardiac hypertrophy following pressure overload. *Physiological reports* **3**, doi:10.14814/phy2.12489 (2015). 10.14814/phy2.12489 PMC4562575 26290533

[25] Gui D (2012). Astragaloside IV, a novel antioxidant, prevents glucose-induced podocyte apoptosis *in vitro* and *in vivo*. PloS one.

[26] Diniz GP, Takano AP, Barreto-Chaves ML (2013). MiRNA-208a and miRNA-208b are triggered in thyroid hormone-induced cardiac hypertrophy - role of type 1 Angiotensin II receptor (AT1R) on miRNA-208a/alpha-MHC modulation. Molecular and cellular endocrinology.

[27] Di Benedetto G, Scalzotto E, Mongillo M, Pozzan T (2013). Mitochondrial Ca(2)(+) uptake induces cyclic AMP generation in the matrix and modulates organelle ATP levels. Cell metabolism.

[28] Arnaiz-Cot JJ (2013). Cardiac calcium signalling pathologies associated with defective calmodulin regulation of type 2 ryanodine receptor. The Journal of physiology.

[29] Cheng KK (2015). Metabolomic analysis of akt1-mediated muscle hypertrophy in models of diet-induced obesity and age-related fat accumulation. Journal of proteome research.

[30] Poulsen L, Siersbaek M, Mandrup S (2012). PPARs: fatty acid sensors controlling metabolism. Seminars in cell & developmental biology.

[31] Drosatos K (2016). Cardiac Myocyte KLF5 Regulates PPARa Expression and Cardiac Function. Circulation research.

[32] Ventura-Clapier R, Garnier A, Veksler V (2004). Energy metabolism in heart failure. The Journal of physiology.

[33] Schilling JD (2015). The mitochondria in diabetic heart failure: from pathogenesis to therapeutic promise. Antioxidants & redox signaling.

[34] Kolwicz SC (2012). Cardiac-specific deletion of acetyl CoA carboxylase 2 prevents metabolic remodeling during pressure-overload hypertrophy. Circulation research.

[35] Liesa M, Shirihai OS (2013). Mitochondrial dynamics in the regulation of nutrient utilization and energy expenditure. Cell metabolism.

[36] Luo M, Anderson ME (2013). Mechanisms of altered Ca(2)(+) handling in heart failure. Circulation research.

[37] Marin-Garcia J, Akhmedov AT, Moe GW (2013). Mitochondria in heart failure: the emerging role of mitochondrial dynamics. Heart failure reviews.

[38] Glancy B, Balaban RS (2012). Role of mitochondrial Ca2+ in the regulation of cellular energetics. Biochemistry.

[39] Li H (2014). Imaging of mitochondrial Ca2+ dynamics in astrocytes using cell-specific mitochondria-targeted GCaMP5G/6s: mitochondrial Ca2+ uptake and cytosolic Ca2+ availability via the endoplasmic reticulum store. Cell calcium.

[40] Penna C, Perrelli MG, Pagliaro P (2013). Mitochondrial pathways, permeability transition pore, and redox signaling in cardioprotection: therapeutic implications. Antioxidants & redox signaling.

[41] Dorn GW (2013). Mitochondrial dynamics in heart disease. Biochimica et biophysica acta.

[42] Kang S (2016). Small Molecular Allosteric Activator of the Sarco/Endoplasmic Reticulum Ca2+ -ATPase (SERCA) Attenuates Diabetes and Metabolic Disorders. The Journal of biological chemistry.

[43] Vejpongsa P, Yeh ET (2014). Wrestling with heart failure: SUMO-1 to the rescue. Circulation research.

[44] Babu GJ, Bhupathy P, Carnes CA, Billman GE, Periasamy M (2007). Differential expression of sarcolipin protein during muscle development and cardiac pathophysiology. J Mol Cell Cardiol.

[45] Kim HK (2015). Echinochrome A regulates phosphorylation of phospholamban Ser16 and Thr17 suppressing cardiac SERCA2A Ca(2)(+) reuptake. Pflugers Archiv: European journal of physiology.

[46] Li Y (2016). CaMKII-dependent phosphorylation regulates basal cardiac pacemaker function via modulation of local Ca2+ releases. American journal of physiology. Heart and circulatory physiology.

[47] Dries E (2016). Calcium/calmodulin-dependent kinase II and nitric oxide synthase 1-dependent modulation of ryanodine receptors during beta-adrenergic stimulation is restricted to the dyadic cleft. The Journal of physiology.

[48] Hu ST (2011). Defective Ca(2+) handling proteins regulation during heart failure. Physiological research.

[49] Gui D (2013). Astragaloside IV ameliorates renal injury in streptozotocin-induced diabetic rats through inhibiting NF-kappaB-mediated inflammatory genes expression. Cytokine.

