# Supplementary material for: Astragaloside IV alleviates heart failure via activating PPARα to switch glycolysis to fatty acid β-oxidation
Source: Sci Rep. 2017 Jun 2;7:2691. doi: 10.1038/s41598-017-02360-5 (PMC5457407; doi:10.1038/s41598-017-02360-5)

Astragaloside IV alleviates heart failure via activating PPARα to switch glycolysis to fatty acid β-oxidation

Zhiwei Dong[1], Pei Zhao[2], Ming Xu[2], Chen Zhang[3], Wei Guo[2], Huihua Chen[2], Jing Tian[2], Hongchang Wei[2], Rong lu[2#], Tongtong Cao[2#]

Supplementary Fig. S1. Original full-length gel images for the Western blotting of PPARα expression in Fig. 2A. Images displayed in this figure are not modified by any image processing software afterimage acquisition and file export. Images displayed in Fig. 2A were cropped from these images but processed and displayed with identical settings.

Supplementary Fig.S1

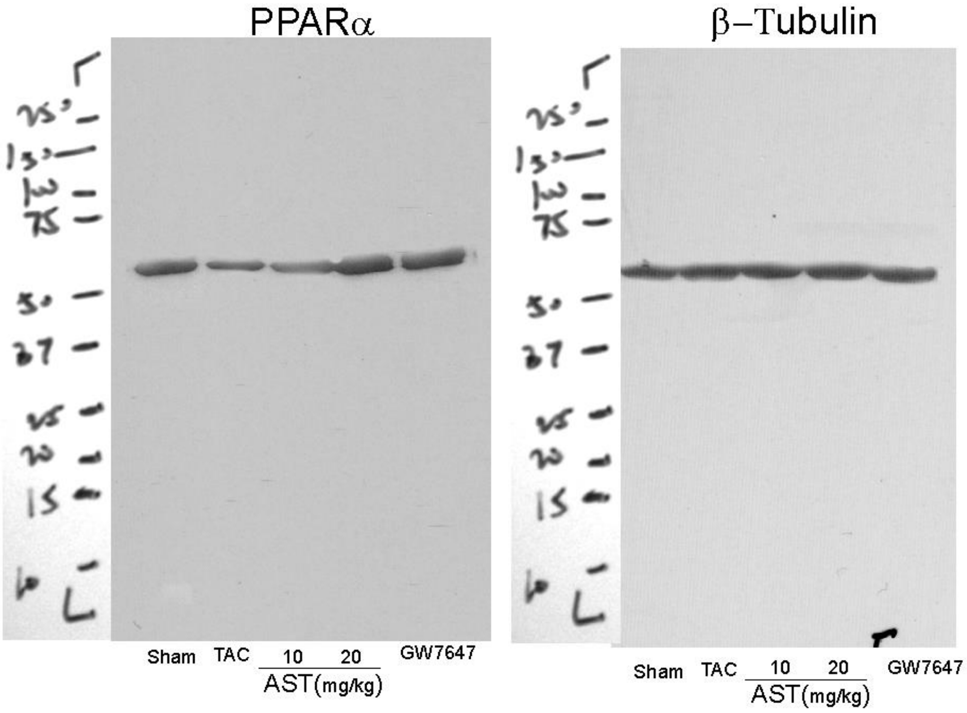

Supplementary Fig. S2. Original full-length gel images for the Western blotting of β-MHC and skeletal α-actin expression in Fig. 3A. Images displayed in this figure are not modified by any image processing software afterimage acquisition and file export. Images displayed in Fig. 3A were cropped from these images but processed and displayed with identical settings.

Supplementary Fig.S2

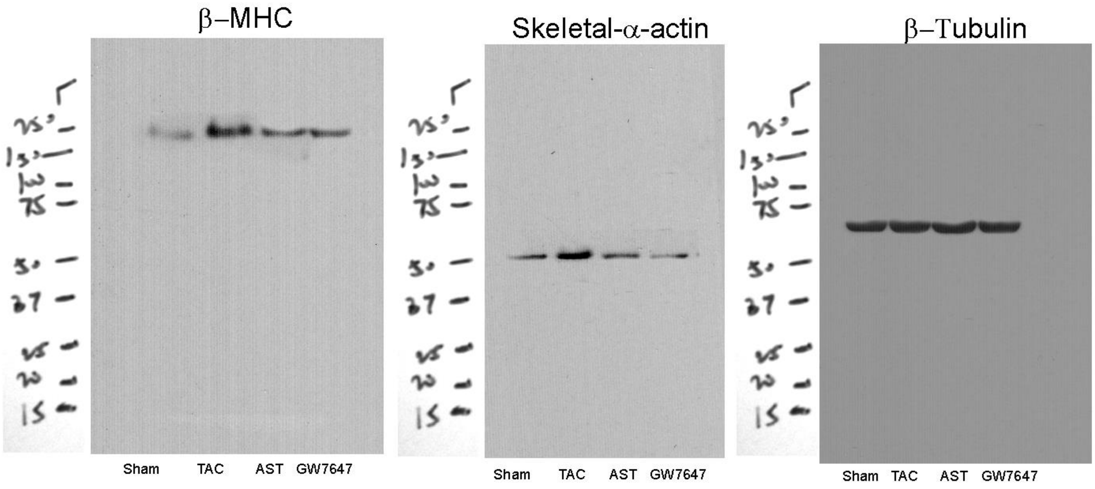

Supplementary Fig. S3. Original full-length gel images for the Western blotting of SERCA2a, NCX, SUMO1, RyR2^{ser2808}, RyR2, PLB^{ser17} and PLB in Fig. 6A. Images displayed in this figure are not modified by any image processing software afterimage acquisition and file export. Images displayed in Fig. 6A were cropped from these images but processed and displayed with identical settings.

Supplementary Fig.S3

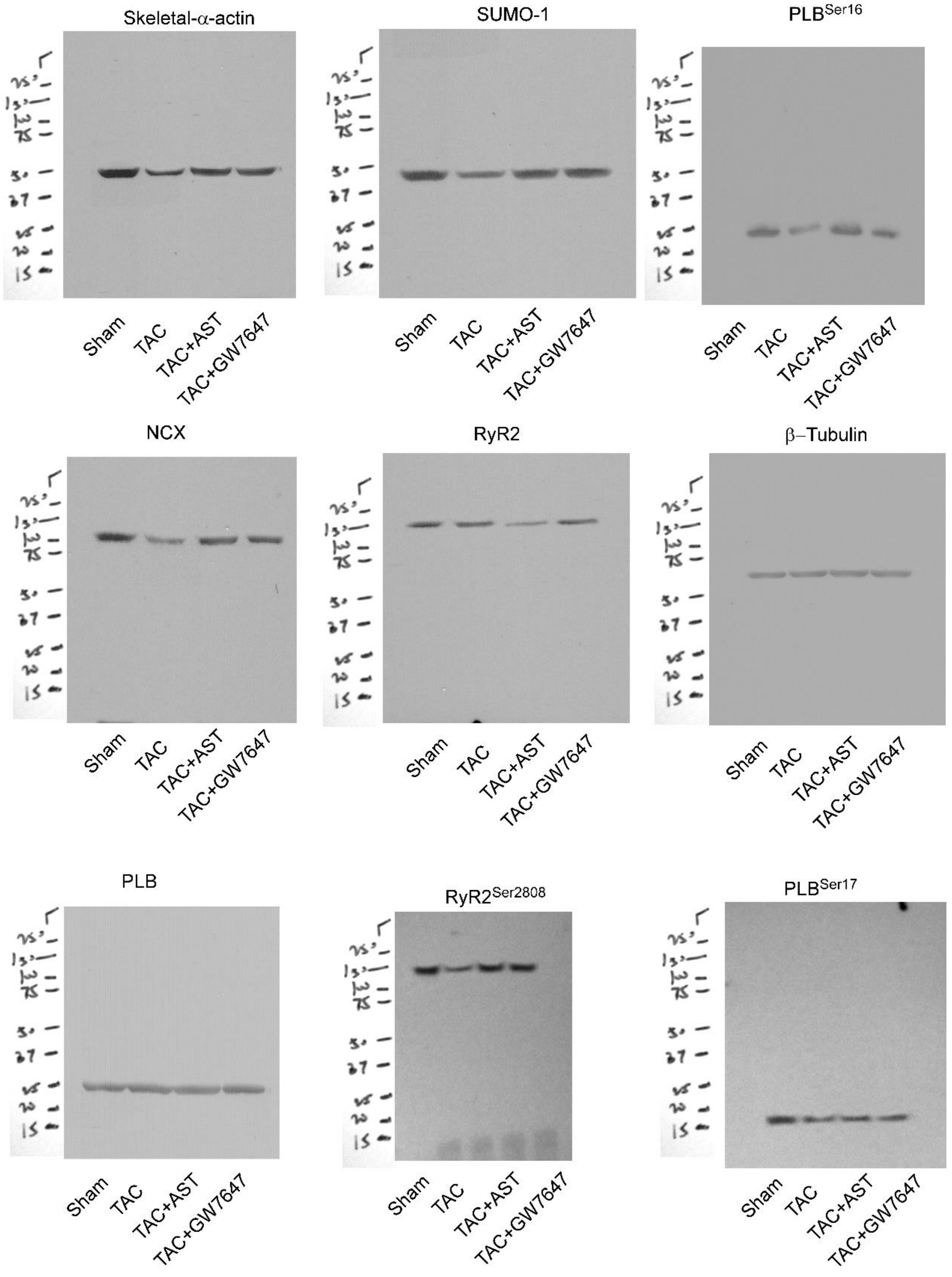

Supplementary Fig. S4. Original full-length gel images for the Western blotting of Bcl-2, Bax, cleaved caspase-3, caspase-3, cleaved PARP and PARP in Fig. 6B. Images displayed in this figure are not modified by any image processing software afterimage acquisition and file export. Images displayed in Fig. 6B were cropped from these images but processed and displayed with identical settings.

Supplementary Fig.S4

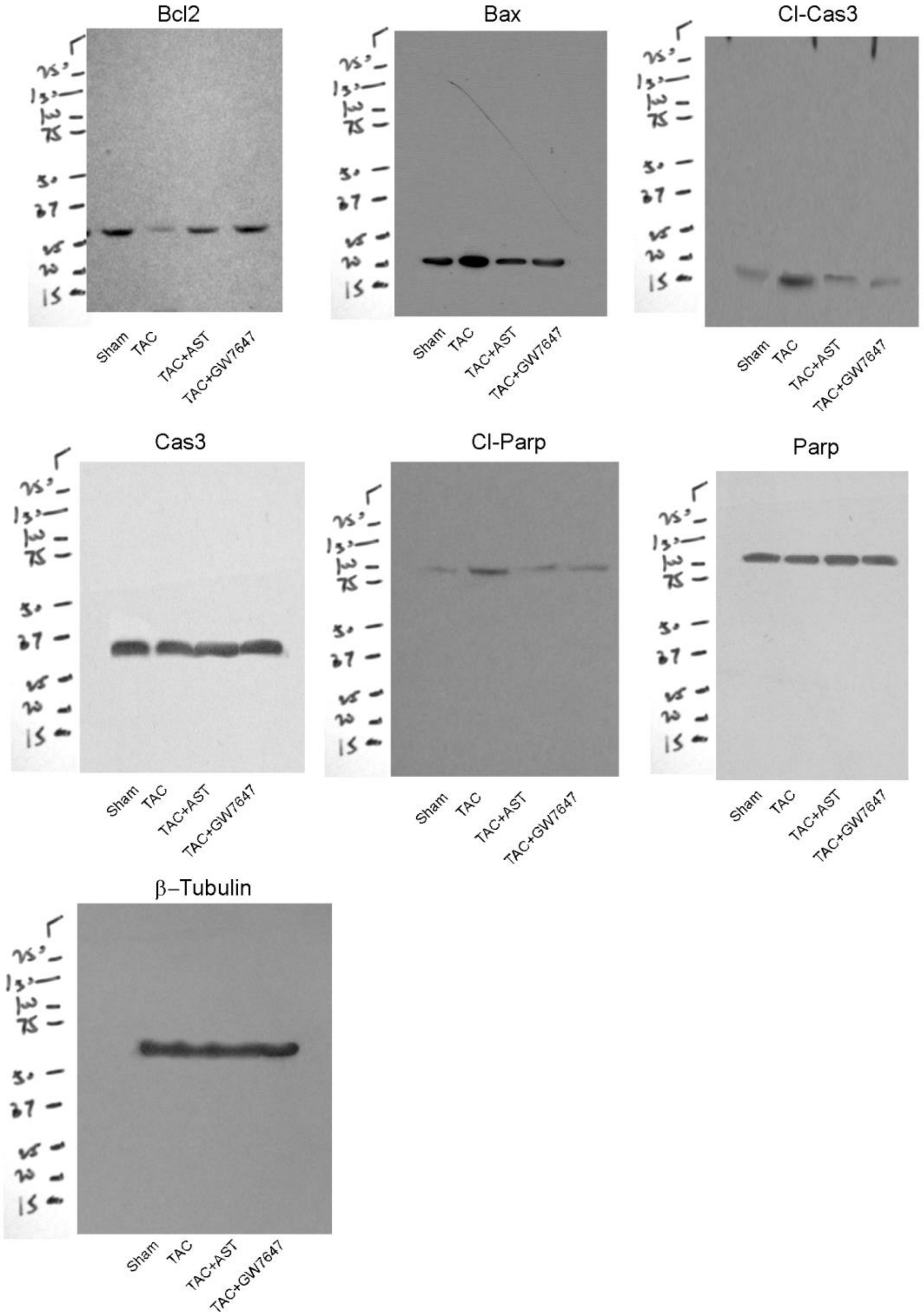

Supplementary Fig. S5. Original full-length gel images for the Western blotting of Glut-4 in Fig. 4D. Images displayed in this figure are not modified by any image processing software afterimage acquisition and file export. Images displayed in Fig. 4D were cropped from these images but processed and displayed with identical settings.

Supplementary Fig.S5

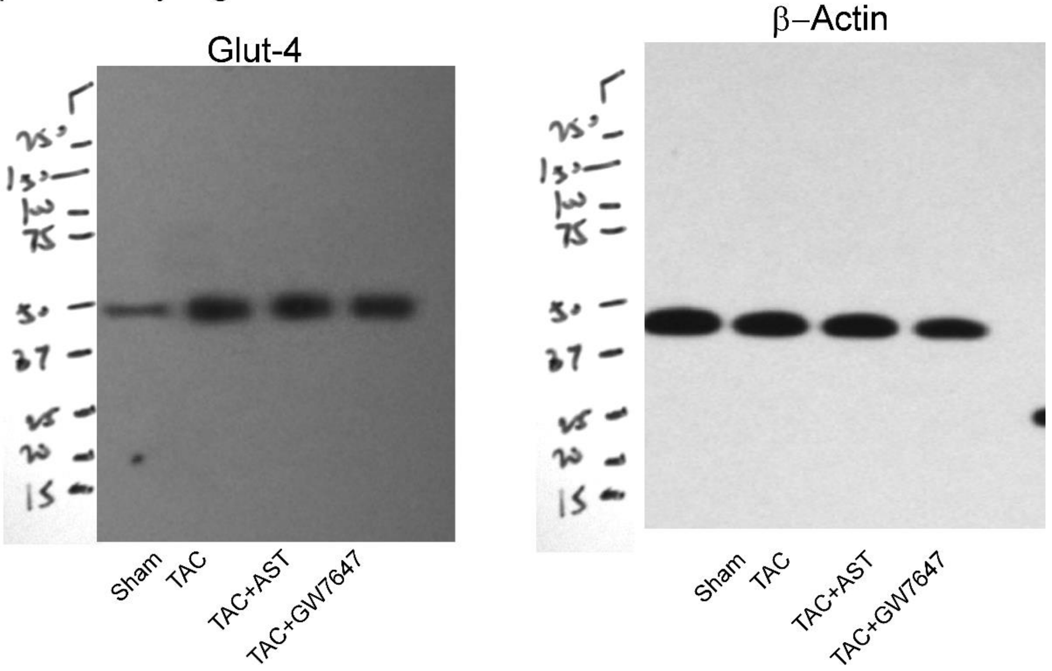

Supplementary Fig. S6. Original full-length gel images for the Western blotting of PPAR- or CyPD expression in Fig.5G. Images displayed in this figure are not modified by any image processing software afterimage acquisition and file export. Images displayed in Fig. 5G were cropped from these images but processed and displayed with identical settings.

Supplementary Fig.S6

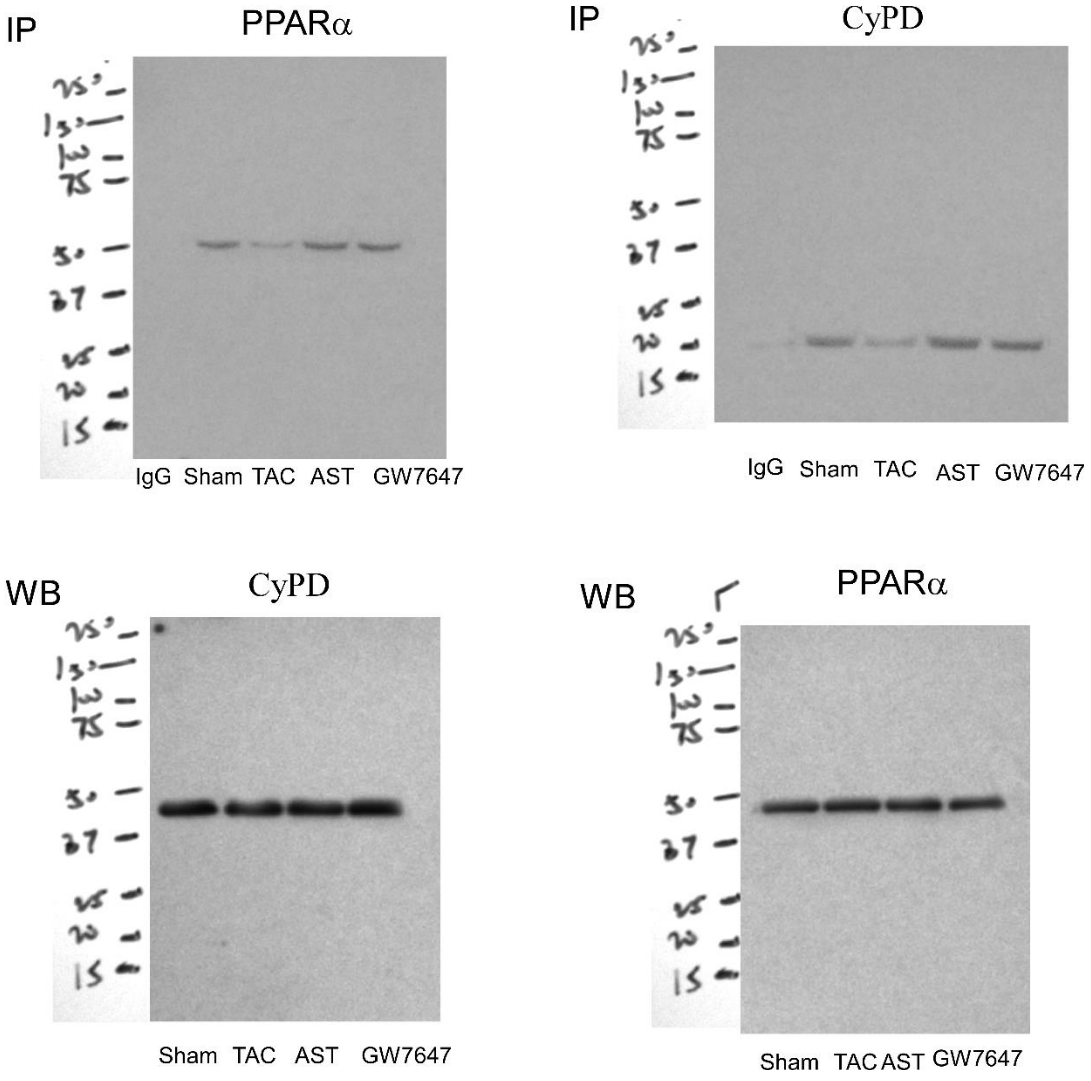

Supplement: Supplementary file 1 — Supplementary data [file 41598_2017_2360_MOESM1_ESM.pdf]
